# Substitution Modeling Shows Simple Dietary Changes Increase Mediterranean-Style Diet Pattern Scores for US Adults

**DOI:** 10.1093/cdn/nzac125

**Published:** 2022-07-23

**Authors:** Francine Overcash, Ambria C Crusan

**Affiliations:** Department of Food Science and Nutrition, University of Minnesota, Minneapolis, MN, USA; Department of Nutrition and Dietetics, St. Catherine University, St. Paul, MN, USA

**Keywords:** Mediterranean diet, adherence, modeling, substitutions, NHANES, adults, honey, dietary pattern, substitutions

## Abstract

**Background:**

A Mediterranean-style diet pattern (MSDP) is a recommended diet pattern in the 2020–2025 Dietary Guidelines for Americans. Few studies report widespread adherence to the diet, which suggests Americans may benefit from strategies to help them improve alignment to an MSDP.

**Objectives:**

The purpose of this study was to assess the impact of isocaloric food substitutions on adherence to an MSDP in US adults.

**Methods:**

Using data from NHANES (2007–2018), alignment to an MSDP was determined by calculation of a Mediterranean diet scoring index appropriate for non-Mediterranean populations (0–100 points for the total score, with higher scores indicating greater adherence). The sample was divided into 2 groups, a high-adherence group (HA) and a non-high-adherence group (nHA), to differentiate food groups to be used for isocaloric food substitution modeling. Substitution modeling via multiple regression analyses determined food selections that have the greatest impact on MSDP scores. Honey was added to the substitutions in recipe form and evaluated for its impact on MSDP scores.

**Results:**

The study consisted of 19,978 adults, ages 25–65, with complete dietary data. The nHA had a lower mean total MSDP score (7.07 ± 0.04 points) compared to the HA (16.45 ± 0.09 points). Increasing olive oil from nonuse to partial use had the greatest impact (>+2 points) for both groups. Other isocaloric substitutions also improved adherence, albeit to a lesser degree, including substituting 1 oz of whole grains for 1 oz of refined grains, 4 oz of fish for 4 oz of red meat, and 4.9 cups of kale for 0.7 cups of starchy or root vegetables. Improved MSDP scores were sustained when honey was added to the substitutions.

**Conclusions:**

Simple dietary substitutions can help a diet more closely align with an MSDP. Adding honey to the simple substitutions may increase palatability without sacrificing nutritional benefits.

## Teaser

This research shows a meaningful improvement in alignment to the Mediterranean Style Diet Pattern is possible with a few simple swaps in their diet.

## Introduction

The vast majority of Americans of all ages fail to meet the recommendations set forth under the Dietary Guidelines for Americans (DGA) (1). The DGA's shift since 2015 in recommending dietary patterns, rather than individual nutrients, foods, or food groups in isolation, is a more accurate depiction of the average American's dietary behaviors and, therefore, is more practical in determining strategies to improve overall dietary intakes.

The 2020–2025 DGA's recommendation of a Mediterranean-style diet pattern (MSDP) is unsurprising given the Mediterranean diet's compelling negative associations with diseases, including cardiovascular disease, diabetes, obesity, Alzheimer's disease, and metabolic syndrome (2–8). The landmark Seven Country Study in the 1950s by Ancel Key and colleagues (9) found strikingly low incidences of coronary heart disease and other chronic diseases among populations in Greece and other Mediterranean regions. Since then, an MSDP has been a dietary pattern of interest.

Willet and colleagues (10) were the first to recommend an MSDP to Americans, in 1995; however, research estimating adherence among Americans is especially limited when removing associations to disease risks (11, 12). That is, MSDP adherence is more commonly the explanatory variable of interest with incidences of diseases or disease risks as the outcome. The current study assesses adherence as the outcome of interest, tested against various other dietary variables as the explanatory factors. The multitude of MSDP adherence scoring indices makes it difficult to determine which methodology is most accurate, especially for non-Mediterranean populations. Often, MSDP scoring systems do not account for overconsumption of certain foods and, therefore, attaining a higher MSDP score (i.e., better adherence) may simply be the result of overconsumption of characteristically MSDP foods, while not accounting for recommended intake amounts (13).

Successful strategies that help non-Mediterranean populations adhere to an MSDP represent a research area with the potential to improve public health nutrition. Research on the foods that characterize intake of MSDP continues to grow in the extant literature. This diet pattern is abundant in plant foods, lean fish, whole grains, legumes, nuts, fresh fruits, vegetables is relatively high in fat from olive oil, low in refined and added sugars, and includes wine in moderation (10). A recent study found online evidence that Americans are confused over what defines the Mediterranean diet, with less than 9% of the online posts studied offering a clear definition (14). Therefore, understanding which foods are readily consumed by Americans and also align with an MSDP may be promising due to the ease of implementation.

Substitution modeling continues to be a growing area of research in nutritional epidemiology. Broadly, various forms of substitution modeling have been used to evaluate the effects of various nutrients on a specific outcome. Ibsen and colleagues (15) provided descriptions of different food substitution modeling methods to assess diet and disease development, ultimately arguing in favor of substitution modeling's potential to inform an optimal food composition of the diet. For example, 1 study found replacing animal-sourced foods with plant-based foods improved nutrient levels, as well as lowered premature mortality (16). Another study reported a reduction in the risk for type 2 diabetes after replacing a serving of processed red meat for an isocaloric serving of poultry (17). A study by Raatz and colleagues (18) used substitution modeling to examine the effect on overall fatty acid intake of replacing high-oleic seed oils for trans fatty acid–containing fats and oils.

The overall goal of the following study is to examine the extents to which dietary substitutions of characteristically MSDP foods may help the average American diet more closely follow an MSDP. The simple dietary substitutions are first identified, then modeled to measure alignment an MSDP using a validated, criterion-based Mediterranean diet scoring index. Substitution modeling has been a tool used in nutritional epidemiology studies to assess how to improve overall dietary patterns (15, 18, 19). Using an MSDP scoring index designed for non-Mediterranean populations (13), we hypothesize our findings will offer empirical evidence that simple dietary swaps can improve MSDP adherence, which has practical implications for those who wish to more closely follow an MSDP. We will examine the effects of the dietary swaps as part of honey-food pairing recipes on MSDP adherence. Honey is a primary sweetener of the Mediterranean region (20), with components and mechanisms relevant for health promotion via anti-oxidant and anti-inflammatory effects (21–23). As such, identification of honey-food pairing recipes represents a novel strategy to make the substitutions more palatable (24) and, in turn, achieve greater widespread MSDP adherence.

## Methods

### Participants and data sets

This cross-sectional analysis used 12 years (2007–2018) of data from the NHANES (25). Since 1999, the CDC has continuously conducted this ongoing, population-based survey, which employs a complex, stratified, multistage probability sample design to create a representative sample of the noninstitutionalized, civilian US population (20). Each survey consists of questionnaires (e.g., sociodemographic, physical activity, sedentary levels, supplement use, etc.) administered in the home by trained personnel, followed by a standardized health examination conducted in person. NHANES dietary data are assessed via two 24-hour recalls. The first recall is conducted in person, with the second collected via phone interview within 3–10 days following the first recall. The final analytic sample consisted of 19,978 adult participants between the ages of 25–65 with two 24-hour diet recalls that were deemed reliable by meeting established criteria by the trained interviewer. Developed by the USDA, the public-use Food Patterns Equivalents Databases (FPED) used in the current study is based on NHANES dietary recall data (26). The FPED uses the USDA food pattern definitions as referenced by the DGA. These food pattern definitions provide calorie-based dietary guidance on how much Americans should eat from each of the food pattern components, such as fruits, vegetables, grains, protein foods, dairy, and oils, to have a healthful diet, while simultaneously placing limits on the amounts of added sugars, solid fats, and alcoholic drinks that can be consumed. The conversion of foods and beverages in the USDA's Food and Nutrient Database for Dietary Studies (27), which provides nutrient compositions of all individual foods and beverages, into 37 USDA food pattern components is reported in the form of food pattern equivalents in the FPED. Many of the foods in the Food and Nutrient Database for Dietary Studies, such as pizza, sandwiches, and casseroles, consist of ingredients that are from more than 1 food pattern component. These multi-ingredient foods are disaggregated to ingredients that can be assigned to a food pattern component before computing the amount of food pattern equivalents present in the food. The food pattern components are subsequently defined as the numbers of cup equivalents of fruit, vegetables, and dairy; ounce equivalents of grains and protein foods; teaspoon equivalents of added sugars; and number of alcoholic drinks, with 1 oz and 1 cup equivalents (eq) translating to 1 serving of each respective food component (26).

### MSDP score

Among the many scoring indices designed to quantify adherence to the Mediterranean diet (28), the current study utilized a scoring index developed by Rumawas et al. (13), as it offered critical advantages over the other criterion-based scoring indexes. This scoring index, called the Mediterranean-style dietary pattern score (MSDP score), accounts for overconsumption of foods and for foods not identified as part of the Mediterranean diet, 2 characterizations of a typical American diet (29).

### Calculation of MSDP score

The MSDP scoring index is based on recommended intakes of 13 food group components from the Mediterranean diet pyramid (10, 13, 30). With the exception of olive oil, scores for the remaining 12 food components are on a continuous scale ranging from 0 to 10, with 10 meaning the recommended intake amount was met per the Mediterranean diet pyramid. First, the average intake by day for each Mediterranean diet food component was calculated by averaging the FPED data across the two 24-hour recalls collected in the NHANES. Next, the component's average intake by day was multiplied by a designated number of points based on recommended intake amounts in the Mediterranean diet pyramid. The 12 Mediterranean diet food components were assigned a specific number of points, so that a maximum score of 10 would be attained if the recommended number of servings per day—or, in the current study's case, the average intake per day—were met. The components were assigned points as follows: whole grains, 1.25 points; fruits, 3.33 points; vegetables, 1.67 points; dairy, 5.00 points; wine for men, 3 points, and wine for women, 1.5 points; fish or seafood, 11.69 points; poultry, 17.5 points; olives, legumes, and nuts, 17.5 points; potatoes and starchy vegetables, 23.31 points; eggs, 23.31 points; sweets and added sugars, 23.31 points; and meat, 70.00 points. For example, if a person consumed 2 servings per day of fruit (2 cup eq) and 2 servings per week of meat, their respective pre-component scores would be 6.66 and 140.00. Overconsumption was then addressed by calculating how much the pre-component score exceeded the recommended amount (as a percentage) and then systematically subtracting a point for each number of servings overconsumed. For example, if a person exceeded their whole grain intake amount by 20%, then the whole grain component score would equal 8. A component score may equal 0 if the intake exceeds the recommendation by >100%, as in the preceding example of consuming 2 servings per week of meat. The olive oil component is measured categorically as exclusive use of olive oil with no other animal fats or vegetable oils used (score of 10), the use of olive oil in combination with other vegetable oils and/or animal fats (score of 5), or no olive oil use and use of other animal fat or vegetable oils exclusively (score 0).

**FIGURE 1:**
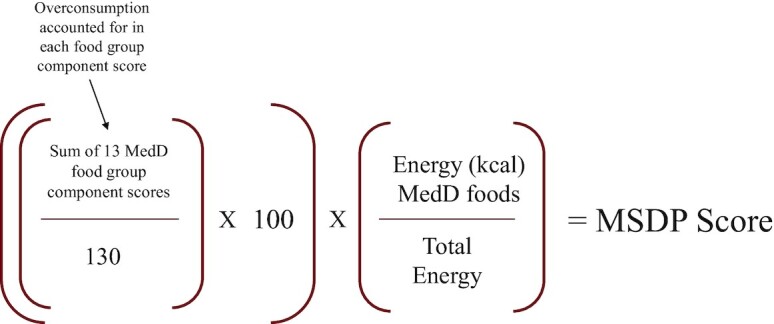
Calculation of MSDP score. MSDP, Mediterranean-style diet pattern.

Scores for all 13 components were summed and standardized to a 0–100 scale by dividing the total sum by 130 (the theoretical maximum sum). This scoring index is tailored for non-Mediterranean populations by using a weighting factor (0–1), to account for typical Mediterranean foods to non-Mediterranean foods. Each food consumed was systematically categorized as an MSDP food compared with a non-MSDP food (13, 31), from which a proportion of the total energy of non-MSDP foods to the total energy of MSDP foods was derived. For example, if a person consumed 60% of their total energy from non-MSDP foods, then the weighting factor would be 0.40. The standardized sum of the 13 components multiplied by the weighting factor is final calculation step. A higher total MSDP score indicates higher adherence to the MSDP (range, 1–100).   **Figure 1** depicts an illustration of the basic steps to calculate the total MSDP score.

### Adherence levels for identification of foods for substitutions

The distribution of total MSDP scores among all participants was examined to create an adherence level categorization variable. Those who fell in quartile 1 (top 25%) of total MSDP scores were categorized as the high-adherence group (HA), while those in quartiles 2–4 constituted the remaining 75% and were categorized as the non-high-adherence group (nHA). The authors hypothesized the food substitutions should come from the food groups where the HA average intakes were both significantly different from the nHA and aligned with the proportions as guided by the Mediterranean diet pyramid. In other words, the foods from these food groups would have the greatest potential to positively impact MSDP scores, especially among the nHA.

The final list of foods to be used as the substitutions were partial use of olive oil, a serving of whole fruit, a serving of whole grain, a serving of fish or seafood, and a serving of nonstarchy vegetables. Next, comparable isocaloric foods among the nHA were identified via calorie matching using the USDA's FoodData Central (32). To determine appropriate isocaloric substitutions, the caloric density of 1 serving of each MSDP food was compared to that of non-MSDP foods within 1 of the food pattern components in the FPED that was overconsumed. For example, to add fish in substitution for red meats, which were both considered protein foods in the FPED, 4 oz of Atlantic salmon is approximately 162 calories and 4 oz of 93% lean ground beef is approximately 172 calories. In a 1-to-1 substitution in which calories were within a small margin between the 2 foods, there were no multipliers applied. However, in the substitutions in which 1 serving was not calorically equal to that of the other item, multiple servings (i.e., 2.5) were applied in the substitution modeling.

### Isocaloric substitution modeling

The current study utilizes an adapted isocaloric substitution modeling process (15, 19, 33) to determine whether simple isocaloric substitutions may help improve adherence to the Mediterranean diet, as measured by the MSDP score (e.g., outcome of interest). The broad concept behind isocaloric substitutions is relatively simple, yet with complex statistical considerations (15, 19): what is the impact on an outcome of interest when an increased intake of a given food is balanced by a decreased intake of another, calorically comparable food? Following identification of the isocaloric pairs of food, systematic calculations ensured individual substitutions would only be applied to participants who met the minimum level of intake for the specific food to be substituted. For example, only those participants who reported consumption of at least 4.0 oz eq of red, processed, or organ meat had 4.0 oz eq of fish or seafood added to their fish or seafood intake amount, balanced by 4.0 oz eq subtracted from their red, processed, or organ meat intake amounts. The presubstitution mean total MSDP score for each adherence group was calculated. For each individual substitution, a postsubstitution mean total MSDP score was calculated and reported by adherence group. The same iterative modeling process was performed, but with multiple isocaloric substitutions added to 1 model to assess for an additive effect on MSDP scores. The simplicity of each individual isocaloric substitution makes it reasonable that multiple isocaloric substitutions may be implemented over a short period of time (e.g., 1 week).

### Substitution modeling using honey-food pairing recipes

**FIGURE 2:**
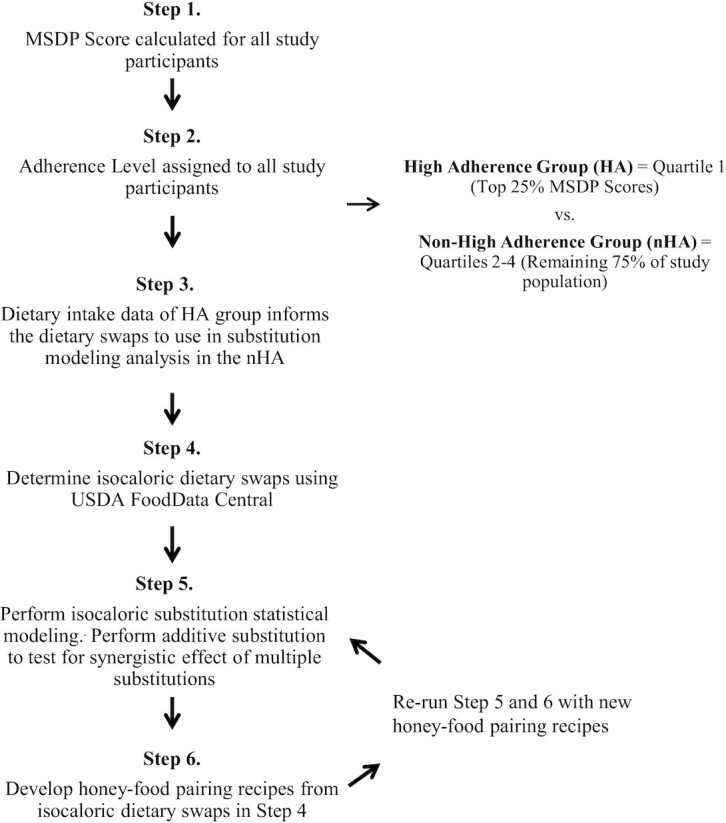
Flowchart of study methods. HA, high-adherence group; LSM, least square means; MSDP, Mediterranean-style diet pattern; nHA, non-high-adherence group.

A substitution analysis using the methods described above assessed the impact on total MSDP scores after adding a serving of honey to the substitutions in the form of recipes. Three easy-to-follow recipes were devised using the 5 isocaloric swaps paired with honey. For example, the substitutions of fruit and a leafy green salad were combined in the recipe of a kale and strawberry salad with olive oil and honey dressing. Each recipe was nutritionally analyzed to determine a reasonable isocaloric swap as described above, but with additional energy (kcal) from the honey factored into the MSDP scoring algorithm as the added sugar and sweets food component (see **Table 1**). To reiterate, the honey-food pairing recipe was substituted if the participant met the minimum level of intake for the specific food(s) to be substituted. In the same example, if the participant had a minimum intake of 2 oz eq of starchy vegetables and 2.45 tsp eq of added sugar in the form of fruit juice, then the substitution recipe of 4 oz of leafy greens and 0.75 cup eq of whole fruit plus the serving of honey (1 tsp of added sugar) would be applied. The flow chart of methods is found in **Figure 2**.

**TABLE 1:** Honey-food pairings and corresponding isocaloric foods^1^

HFP description	Nutritional information of HFP	Comparable ISF	Nutritional information of comparable ISF
HFP 1: strawberry kale salad with olive oil and honey dressing swapped for starchy vegetable	• 0.75 cups (114 g) of strawberries provide 39.9 calories and 8.6 g of carbohydrate: 0 tsp eq of added sugar	ISF 1 foods/description	• 3.6 oz apple juice from concentrate (100 g) provides 48 calories and 11.4 g carbohydrate: 2.45 tsp eq of added sugar
	• 4.9 cups of kale (100.9 g) provide 43.4 calories and 4.4 g of carbohydrate	—	• 0.33 cups (50 g) of boiled potato provides 43.5 calories and 10 g of carbohydrate
	• 0.5 tbsp (7 g) of olive oil provide 59 calories and 6.6 g of fat	—	• 0.5 tbsp (7 g) of unsalted butter provides 51.4 calories and 5.7 g of fat
	• 0.33 Tbsp honey provides 21.1 calories and 1 tsp eq added sugar		
HFP 2: Tabouleh with olive oil or honey dressing swapped for refined grain	• 0.5 cups (92.5 g) of cooked quinoa provides 111 calories and 19.7 g of carbohydrate	ISF 2 foods/description	• 0.5 cups (93 g) of white rice contains 121 calories and 26.6 g of carbohydrate
	• 2 tbsp (28 g) of olive oil provides 236.2 calories and 26.2 g of fat	—	• 2 tbsp (28 g) of unsalted butter provides 136.7 calories and 15.2 g of fat
	• 0.4 cups (41.25 g) of nonstarchy veggies (parsley, carrot) provides 15.2 calories	—	—
	• 0.75 tbsp of honey provides 47.9 calories and 3 tsp eq of added sugar	—	—
HFP 3: Fish with olive oil or honey marinade swapped for red meat	• 4 oz (113.3 g) of Atlantic salmon provides 161.3 calories, 22.4 g of protein, and 7.2 g of fat	ISF 3 foods/description	• 4 oz (113 g) of 93% lean ground beef provides 172 calories, 23.5 g of protein, and 7.9 g of fat
	• 1.33 tbsp (18.6 g) of olive oil provides 156.9 calories and 17.6 g of fat	—	• 1.33 tbsp (18.6 g) of canola oil provides 158.4 calories and 22.8 g of fat
	• 0.33 tbsp of honey provides 21.1 calories and 1 tsp eq of added sugar	—	—

1 eq, equivalents; HFP, honey-food pairing; ISF, isocaloric foods.

### Statistical analysis

The data analysis was conducted using SAS v. 9.4 (34) Survey Procedures with NHANES-supplied sampling weights to account for the complex, multistage, probability sampling design of NHANES data. Differences by adherence group across demographic and BMI status characteristics were determined by a chi-square test using PROC SURVEYFREQ. The following characteristics that significantly differed were included as cofactors in the multiple regression models: age, gender, race, education, and BMI. Multiple regression analyses using PROC SURVEYREG determined differences by adherence group for the following continuous dietary outcome variables: total MSDP score, the 12 component scores, and average intake per day amounts (cup or ounce equivalents). The regression models examining average intakes per day also adjusted for daily energy (kcal). Because olive oil is a categorical variable, a chi-square test using PROC SURVEYFREQ was applied to determine the differences in olive oil use between adherence groups. Multiple regression analyses, adjusted for age, sex, race, education level, and BMI using PROC SURVEYREG, were used to determine the impacts of individual isocaloric substitutions on total MSDP scores for both adherence groups. A Student *t*-test was used to compare the change from pre-to postsubstitution between adherence groups. Paired *t*-tests were performed to detect within-group changes (i.e., changes from presubstitution to postsubstitution MSDP scores). Multiple regression analyses, adjusted for age, sex, race, education level, and BMI using PROC SURVEYREG, were also used to assess the effects of multiple isocaloric substitutions (i.e., additive models, with and without honey) on mean total MSDP scores for both adherence groups. All reported *P* values were 2-tailed and considered statistically significant at a *P* value < 0.05.

NHANES is conducted according to guidelines set forth by the Declaration of Helsinki, and all procedures involving human subjects were approved by the National Center for Health Statistics Ethics Review Board (35).

## Results

### Sociodemographic characteristics and BMI

Compared to the nHA, more HA were female, 50+ years old, and had attained a college degree or higher. The prevalence of a healthy or underweight BMI was higher in the HA compared to the nHA (31% HA compared with 25% nHA in the healthy and underweight categories; 68% HA compared with 74% nHA in the overweight and obese categories; **Table 2**). All characteristics differed by adherence level (all *P* values < 0.0001).

**TABLE 2:** Sociodemographic characteristics and BMI statuses by MSDP scores^1^

Characteristic	HA (*n* = 4452)	nHA (*n* = 15,526)
	Frequency (%)	Frequency (%)
Sex		
Male	1773 (40)	7751 (50)
Female	2679 (60)	7775 (50)
Age, y		
<30	372 (8)	1889 (12)
30–39	970 (22)	3845 (25)
40–49	1058 (24)	3748 (24)
50–59	1156 (26)	3575 (23)
≥60	896 (20)	2469 (16)
Race and ethnicity		
Mexican or other Hispanic	1139 (26)	4267 (27)
Non-Hispanic white	1739 (39)	5844 (38)
Non-Hispanic black	873 (20)	3576 (23)
Multiple races or other	701 (16)	1839 (12)
Education level		
<12th grade, high school graduate, or GED	1427 (32)	7391 (48)
Some college or Associate's degree	1231 (28)	4709 (30)
College graduate or higher	1792 (40)	3414 (22)
BMI status		
Underweight and healthy	1361 (31)	3915 (25)
Overweight	1439 (32)	4972 (32)
Obese	1622 (36)	6525 (42)

1 HA compared with nHA in adults 25–65 years in NHANES 2007–2018: For all sociodemographic charactestics and BMI status, attained statistical significance at *P* < 0.0001, using Rao-Scott *F*–adjusted chi-square statistic test. GED, General Educational Development Test; HA, high-adherence group; MSDP, Mediterranean-style diet pattern; nHA, non-high-adherence group.

### Total MSDP scores and average intakes of food group components

The mean total MSDP score for the HA (16.45 ± 0.09 points) was more than twice that of the nHA (7.07 ± 0.04 points; *P* value < 0.0001; **Table 3**). The HA had significantly greater average intakes per day in cup or ounce equivalents for whole grains, total fruits, nonstarchy vegetables, dairy, wine, fish or seafood, and nuts or legumes, while the added sugar intake was lower compared to the nHA (all *P* values < 0.0001 - not shown;   Table 3). The percentage of HA who reported use of olive oil (partial and only) was over 6 times greater than the same percentage of nHA (*P* value < 0.0001; **Table 4**). Table 3 also presents the MSDP score components, which are based on the average intake amounts per day and are reflective of overconsumption. The differences in magnitude of the average intake results from Table 3 directly informed the isocaloric substitutions listed in **Table 5**.

**TABLE 3:** MSDP average intake amounts and score components for continuous variables^1^

	HA (*n* = 4452)		nHA (*n* = 15,526)	
	Average intake per day LSM (SE)^2^	MSDP component score^3^ (out of 10)	Average intake per day LSM (SE)^2^	MSDP component score^3^ (out of 10)
Total MSDP score^4^	16.45 (0.09)	—	7.07 (0.04)	—
Whole grains^4^	1.44 oz eq (0.06 oz eq)	1.8 (0.05)	0.65 oz eq (0.04 oz eq)	0.79 (0.02)
Total fruits^4^	1.50 cup eq (0.05 cup eq)	4.27 (0.07)	0.74 cup eq (0.04 cup eq)	2.16 (0.04)
Nonstarchy vegetables^4^	1.44 cup eq (0.04 cup eq)	2.38 (0.05)	1.00 cup eq (0.04 cup eq)	1.67 (0.02)
Dairy^4^	1.61 cup eq (0.05 cup eq)	5.91 (0.07)	1.51 cup eq (0.05 cup eq)	4.56 (0.04)
Wine^4^	0.33 glasses (0.03 glasses)	1.16 (0.07)	0.11 glasses (0.02 glasses)	0.27 (0.02)
Fish and other seafood^4^	0.84 oz eq (0.06 oz eq)	1.58 (0.07)	0.57 oz eq (0.05 oz eq)	0.61 (0.02)
Poultry	1.65 oz eq (0.08 oz eq)	1.73 (007)	1.55 oz eq (0.07 oz eq)	0.90 (0.03)
Nuts and legumes^4^	1.11 oz eq (0.08 oz eq)	3.17 (0.09)	0.74 oz eq (0.09 oz eq)	1.96 (0.04)
Starchy root vegetables	0.44 cup eq (0.02 cup eq)	4.32 (0.09)	0.45 cup eq (0.20 cup eq)	2.60 (0.05)
Eggs	0.55 oz eq (0.03 oz eq)	2.99 (0.07)	0.57 oz eq (0.03 oz eq)	2.03 (0.03)
Added sugars^4^	14.89 tsp eq (0.24 tsp eq)	0.14 (0.02)	18.41 tsp eq (0.21 tsp eq)	0.04 (0.01)
Meat (red, organ, processed)	2.66 oz eq (0.10 oz eq)	0.39 (0.04)	2.74 oz eq (0.09 oz eq)	0.17 (0.01)

1 HA compared with nHA in adults 25–65 years in NHANES 2007–2018. eq, equivalents; HA, high-adherence group; LSM, least square means; MSDP, Mediterranean-style diet pattern; nHA, non-high-adherence group.

2 Average intake per day was averaged from 2 dietary recalls. The regression models were adjusted for age, sex, race, education level, BMI, and calories.

3 The regression models were adjusted for age, sex, race, education level, and BMI. The closer a score is to 10, the closer it is to the recommended intake levels per the Mediterranean diet pyramid.

4 HA compared with nHA average intake per day: attained statistical significance at *P* < 0.0001, using multiple regression.

**TABLE 4:** MSDP average intake amounts and score components for categorical variables^1^

	Olive oil use^2^		
	Only olive oil, frequency (%)	Olive oil + other vegetable oils and animal fats, frequency (%)	No olive oil use
HA	93 (3.04)	139 (3.62)	4220 (93.33)
nHA	46 (0.30)	58 (0.42)	15,422 (99.28)

1 HA compared with nHA in adults 25–65 years in NHANES 2007–2018. HA, high-adherence group; MSDP, Mediterranean-style diet pattern; nHA, non-high-adherence group.

2 HA compared with nHA: attained statistical significance at *P*< 0.0001, using Rao-Scott *F*–adjusted chi-square statistic test.

**TABLE 5:** Effects of individual isocaloric substitutions on MSDP scores^1^

Isocaloric substitutions	HA^2^ (*n* = 4452)			nHA^3^ (*n* = 15,526)			*P* value^4^
	Mean MSDP score, LSM ± SE (95% CI)	*P* value^5^	% of HA substitution applied	Mean MSDP score LSM ± SE (95% CI)	*P* value^5^	% of nHA substitution applied	
Substitution 1: partial olive oil use for no olive oil use	19.02 ± 0.09 (18.84, 19.19)	<0.0001	95%	9.10 ± 0.04 (9.00, 9.19)	<0.0001	99%	<0.0001
Substitution 2: whole fruit (0.75 cup eq) for fruit juice (2.45 added sugar tsp eq)	17.36 ± 0.10 (17.17, 17.55)	<0.0001	92%	7.99 ± 0.04 (7.90, 8.07)	<0.0001	94%	<0.0001
Substitution 3: whole grain (1 oz eq) for refined grain (1 oz eq)	17.06 ± 0.09 (16.88, 17.23)	<0.0001	95%	7.54 ± 0.04 (7.45, 7.62)	<0.0001	97%	<0.0001
Substitution 4: leafy greens (4.9 cup eq) for starchy vegetable (2 cup eq)	17.38 ± 0.11 (17.17, 17.59)	<0.0001	17%	7.19 ± 0.04 (7.10, 7.28)	<0.0001	25%	<0.0001
Substitution 5: fish or seafood (4 oz eq) for red or processed meat (4 oz eq)	16.37 ± 0.09 (16.19, 16.54)	<0.0001	19%	7.08 ± 0.04 (7.00, 7.16)	<0.0001	25%	<0.0001

1 HA compared with nHA in adults 25–65 years in NHANES 2007–2018. The regression models were adjusted for sex, age, race, education, and BMI. eq, equivalents; HA, high-adherence group; LSM, least square means; MSDP, Mediterranean-style diet pattern; nHA, non-high-adherence group.

2 Presubstitution mean MSDP score ± SE, 16.45 ± 0.09 (95% CI: 16.27, 16.62).

3 Presubstitution mean MSDP score ± SE, 7.07 ± 0.04 (95% CI: 6.99, 7.15). *P* < 0.0001. when comparing presubstitution scores between HA and nHA.

4 Between-group (HA compared with nHA) *t*-test *P* values compare changes from presubstitution to postsubstitution mean MSDP scores.

5 Within-group paired *t*-test *P* values compare changes from presubstitution to postsubstitution mean MSDP scores.

### Isocaloric substitutions and MSDP scores

The 5 isocaloric substitutions assessed in the current study are found in Table 5. Substituting partial use of olive oil for those who did not report any use had the greatest effect on MSDP scores for both adherence groups (HA score difference: 2.6 points, an ∼16% increase from the presubstitution score; nHA score difference: 2.0 points, an ∼28% increase from presubstitution score), which is unsurprising given the substitution applied to 95% and 99% of the HA and nHA, respectively. Replacing fruit juice, in the form of 2.45 tsp eq of added sugars for 0.75 cup eq of whole fruit, resulted in an ∼5% increase (+0.9 points) from the presubstitution score for the HA and an ∼13% increase (+0.9 points) from the presubstitution score for the nHA. Replacing 1 oz eq of refined grain for 1 oz eq of whole grain also increased MSDP scores for both groups, with an ∼4% increase (+0.6 points) for the HA and an ∼7% increase (+0.5 points) for the nHA. Both substitutions applied to most of the study population (over 90% for both adherence groups). Replacing 0.67 cup eq of starchy vegetables for 4.9 cup eq of leafy greens had a greater impact on the HA compared with nHA [∼6% increase (+ 0.9 points) compared with 2% increase (+0.1 points) from presubstitution to post-substitution MSDP scores, respectively] and applied to smaller percentages of both groups (17% of the HA and 25% of the nHA). The effects of the isocaloric substitution of fish or seafood for red, processed, and/or organ meat had the smallest effects on the MSDP scores for both groups. Although very small in magnitude, the effect remained positive for the nHA (difference: 0.01 points, an ∼0.1% increase from presubstitution score), unlike the decrease in score found for the HA. All 5 individual substitutions significantly improved MSDP scores from their respective presubstitution scores for the nHA (all *P* values < 0.0001;   Table 5).

### Additive modeling of isocaloric substitutions and MSDP scores

In general, the mean MSDP scores increased with each new substitution added to the model (all within-group *t*-test *P* values < 0.0001). Thus, a cumulative effect is suggested for both adherence groups, although it is more pronounced in the nHA. Using the results from Table 5, olive oil was used as the base substitution (**Table 6**). When the whole fruit substitution was added to the olive oil substitution, MSDP scores increased by 3.4 points (∼21% increase) for the HA and 2.9 points (∼41% increase) for the nHA. Although the MSDP scores generally improved with each addition, each additional substitution decreased in magnitude of improvement compared to the preceding model. The model that included all 5 substitutions improved the MSDP score by just 0.02 points above the model with 4 substitutions for the nHA. This was not the case for the HA, where the model decreased the total score by 0.09 points. The score change for every addition was significantly different from the presubstitution score (all *P* values < 0.0001;   Table 6).

**TABLE 6:** Effects of additive modeling of isocaloric substitutions on MSDP scores^1^

Substitutions included in model	HA (*n* = 4452)^2^		nHA (*n* = 15,526)^3^		*P* value^4^
	LSM ± SE (95% CI)	*P* value^5^	LSM ± SE (95% CI)	*P* value^5^	
Substitutions 1 and 2	19.82 ± 0.09 (19.63, 20.00)	<0.0001	9.95 ± 0.05 (9.85, 10.05)	<0.0001	<0.0001
Substitutions 1, 2, and 3	20.42 ± 0.09 (20.24, 20.61)	<0.0001	10.42 ± 0.05(10.32, 10.51)	<0.0001	<0.0001
Substitutions 1, 2, 3, and 4	20.52 ± 0.09 (20.33, 20.71)	<0.0001	10.53 ± 0.05 (10.43, 10.64)	<0.0001	<0.0001
All 5 substitutions	20.43 ± 0.09 (20.25, 20.62)	<0.0001	10.55 ± 0.05 (10.44, 10.65)	<0.0001	<0.0001

1 HA compared with nHA in adults 25–65 years in NHANES 2007–2018. The regression models were adjusted for sex, age, race, education, and BMI. Substitution 1 is partial olive oil use for no olive oil use; substitution 2 is whole fruit (0.75 cup eq) for fruit juice (2.45 cup eq); substitution 3 is whole grain (1 oz eq) for refined grain (1 oz eq); substitution 4 is leafy greens (4.9 cup eq) for starchy vegetables (2 cup eq); and substitution 5 is fish or seafood (4 oz eq) for red or processed meat (4 oz eq). eq, equivalents; HA, high-adherence group; LSM, least square means; MSDP, Mediterranean-style diet pattern; nHA, non-high-adherence group.

2 Presubstitution mean MSDP score ± SE, 16.45 ± 0.09 (95% CI: 16.27, 16.62).

3 Presubstitution mean MSDP score ± SE, 7.07 ± 0.04 (95% CI: 6.99, 7.15). *P* < 0.0001

when comparing presubstitution scores between HA and nHA.

4 Between-group (HA compared with nHA) *t*-test *P* values compare changes from presubstitution to postsubstitution mean MSDP scores.

5 Within-group paired *t*-test *P* values compare presubstitution to postsubstitution MSDP scores.

### Effects of isocaloric honey-food pairings on MSDP scores

The term “honey-food pairings” describes easy-to-follow recipes cultivated from the isocaloric substitutions with the addition of honey. Table 1 lists the nutritional information for both the honey-food pairings and comparable foods to be replaced in the isocaloric substitution models. Using the results from the preceding isocaloric substitutions, 3 honey-food pairing recipes were tested. It should be noted that after using the USDA's FoodCentral database, the comparable isocaloric foods to be substituted did not change from the analysis without honey, despite the added sugars honey brings to each recipe. Each honey-food pairing recipe significantly improved mean MSDP scores for both adherence groups (changes in scores from presubstitution; all *P* values < 0.0001), with the improvements more pronounced for the nHA (**Table 7**). The tabbouleh honey-food pairing recipe had the most positive effect, with an ∼21% increase (+3.5 points) for the HA and a 38% increase (+2.7 points) for the nHA, followed by the leafy green with olive oil and honey dressing honey-food pairing recipe (HA: +2.7 points; nHA: +2.2 points). The recipe for fish with a honey, herb, and olive oil marinade had the smallest positive impact (HA: +2.5 points; nHA: +2.0 points). A cumulative effect was seen when all 3 honey-pairing substitutions were included in the model, with a 23% increase (+3.9 points) for the HA and an ∼49% (+3.5 points) increase for the nHA.

**TABLE 7:** Effects of HFP isocaloric substitutions (individual and cumulative) on MSDP scores^1^

Isocaloric HFP recipe substitutions	HA^2^ (*n* = 4452)		nHA^3^ (*n* = 15,526)		*P* value^4^
	LSM ± SE (95% CI)	*P* value^5^	LSM ± SE (95% CI)	*P* value^5^	
HFP 1: Strawberry kale salad with olive oil or honey dressing swapped for starchy vegetable	19.11 ± 0.09 (18.94, 19.29)	<0.0001	9.23 ± 0.05 (9.14, 9.33)	<0.0001	<0.0001
HFP 2: Tabouleh with olive oil or honey swapped for refined grain	19.89 ± 0.09 (19.71, 20.07)	<0.0001	9.84 ± 0.05 (9.71, 9.90)	<0.0001	<0.0001
HFP 3: Fish with honey marinade swapped for red meat	18.92 ± 0.09) (18.75, 19.10)	<0.0001	9.10 ± 0.05 (9.02, 9.20)	<0.0001	<0.0001
All 3 HPFs	20.34 ± 0.10 (20.15, 20.54)	<0.0001	10.53 ± 0.05 (10.42, 10.63)	<0.0001	<0.0001

1 HA compared with nHA in adults 25–65 years in NHANES 2007–2018. The regression models were adjusted for sex, age, race, education, and BMI. HA, high-adherence group; HFP, honey-food pairing; LSM, least square means; MSDP, Mediterranean-style diet pattern; nHA, non-high-adherence group.

2 Presubstitution mean MSDP score, 16.45 (SE 0.09).

3 Presubstitution mean MSDP score, 7.07 (SE 0.04). *P* < 0.0001 when comparing presubstitution scores between HA and nHA.

4 Between-group (HA compared with nHA) t-test *P* values compare changes from presubstitution to postsubstitution mean MSDP scores.

5 Within-group paired *t*-test *P* values compare presubstitution to postsubstitution MSDP scores.

## Discussion

The current study offers evidence that the vast majority of American adults fall short in following an MSDP, despite it being 1 of 3 dietary patterns recommended in the last 2 iterations of the DGA. The popularity of an MSDP, as indicated by the large number of reports that span both science and current news publications (36–39), presents a conundrum for public health nutrition when juxtaposed against our findings. The present study provides empirical data in support of a strategy that uses simple substitutions the average American can implement in their eating habits to help increase their adherence to an MSDP.

The current study found even lower adherence to an MSDP in comparison to the few studies among US populations in the extant literature. Rumawas et al. (13) determined greater adherence (mean score: 24.8) using the same scoring system in a cohort of Americans whose previous generation(s) participated in the Framingham Heart Study. As such, selection bias may have been present, because participating offspring may have been informed of a family history of cardiovascular disease or related chronic conditions and, therefore, may have already been following a more heart-healthy dietary pattern. Unlike the nationally representative sample used in the current study, the Framingham Heart Study is based in the northeastern region of the United States. Chen et al. (11) found that 46.5% of their study participants had high adherence (scoring from 5–9 out of 9 points) using an FFQ among 20,897 Americans. The top 25% of scorers in the current study had a mean score of 16.45 (out of 100). Direct comparison to the Chen et al. (11) study proves problematic given the significant differences in study methodologies. For example, data were assessed in the Chen et al. (11) study using an MSDP scoring system that did not mention accounting for overconsumption of “detrimental” foods (i.e., added sugars). Our findings provide clear evidence that Americans have patterns of overconsumption, especially in the non-high-adherence group, that may negate the effects of achieving the desired quantity of “beneficial” foods in an MSDP. The Chen et al. (11) cohort was also restricted to those ≥45 years of age. Older Americans have been found to have healthier dietary patterns in previous ecological studies (40). Finally, the information obtained from 24-hour recalls, compared to FFQs, has been found to more closely align with intake as measured by the doubly labeled water method (41).

In assessing adherence to an MSDP, the current study offers supplemental guidance to the DGA to show how much Americans continue to overconsume certain food groups (42). Considering a score of 10 for each food group equates meeting the recommend MSDP amounts, the HA and nHA mean scores for meat (0.39 ± 0.04 and 0.17 ± 0.01, respectively) and added sugars (0.14 ± 0.02 and 0.04 ± 0.01, respectively) illustrate a critical need for improvement. These numbers translate to alarming overconsumption, especially for the nHA. Similar results were previously reported, with 91.4% of participants exceeding the recommendations for meat and 90.7% overconsuming added sugar (13). Our results also show poultry and eggs are popularly consumed protein sources in American diets, but not to the same degrees of overconsumption as red meats and added sugars. Industry data support these findings, reporting average per capita consumption of poultry between 2007–2018 at 102.1 pounds (43) and a 15% increase in per capita egg consumption since 2007 (44). The mean scores for nuts and legumes were also indicative of overconsumption, at 3.17 ± 0.09 in the HA and 0.74 ± 0.09 in the nHA, although not to the degrees relative to meat and added sugars. This could be attributed to the growth in popularity of plant-based diets (45) and the wider availability of plant-based products in the market (46). Future interventions to increase MSDP adherence can use the present study's results to inform strategies to reduce intakes for certain food groups.

Less than ideal average intakes per day were also present for other MSDP food groups, including whole grains, nonstarchy vegetables, fish, and olive oil. When juxtaposed against a 36% increase in whole grains in US food products since 2008 (47), whole grain intakes are low, especially among the nHA, with reported consumption of 0.65 oz eq per day (MSDP component score = 0.79 out of 10). The 75% of the study population making up the nHA achieved a total vegetable intake of ∼1 cup eq, which is lower than the 2–3 cups per day recommended by the DGA. Lee et al. (48) found an even smaller proportion of their study population (10%) met the same DGA benchmark, making the 6 cups per day recommendation of the MSDP even more challenging to achieve. Fruit consumption fared only slightly better among the HA, whose average intake (1.50 ± 0.05 cup eq) was within range of meeting the 1.5–2 cup recommendation set by the DGA (39) but still fell short of the 3-cup MSDP recommendation. The proportion of those who reported any use of olive oil in the HA was much greater than that in the nHA (6.8% compared with 0.7%, respectively). Rumawas and colleagues (13) found that 37% of their study population exclusively used olive oil, thereby dwarfing the current study's reported ∼3.3% of the total population for the same level of olive oil use. The differentiation is likely attributable to the difference in data collection of a 24-hour recall compared with an FFQ. Careful consideration needs to be applied when measuring olive oil use in future research. The aforementioned food groups are emphasized in an MSDP and are, therefore, critical to include in the diet substitutions. The wine intake results suggested a favorable contribution to the average American's MSDP adherence. However, the authors focused only on foods for the substitutions, considering wine is a limited MSDP item (10).

Using foods that are already part of the average American diet underscores widespread acceptability to elicit behavior change. That is, first determining which foods differed between high and lower adherence groups clarifies which foods may be most predictive of improved alignment and, thus, optimal for the substitutions. A recent study assessing factors that determine high compared with low adherence to dietary prescriptions noted participants were more likely to continue to make dietary substitutions to nutrient-dense options 6 months after a diet intervention than they were to follow a diet prescription (49). Further research would need to confirm whether these foods have the potential to be accepted by the broader population. The substitution that made the greatest impact on MSDP scores was increasing olive oil use, which makes sense given the vast majority of the study population (98%) reported no olive oil use. Research widely supports exclusive olive oil use as the most representative component of the MSDP, as well as the most impactful component for cardiovascular health (50) and successful aging (51). This simple substitution increased total mean MSDP score for the nHA by over 2 points, and over 2.5 points for the HA, as shown in Table 5." THEN move this revised sentence before Research widely supports exclusive olive oil use as the most representative. The finding that the fish or seafood substitution did not positively impact the MSDP score suggests the HA was closer to meeting the ideal number of servings. Practicing nutritionists and clinicians may find this information helpful in tailoring substitutions for their patients.

The effects of the number of dietary substitutions on the MSDP scores provided compelling results with practical implications for dietary behavior changes. An additive effect on MSDP scores was shown with multiple substitutions. The ease and practicality of 1–3 simple food swaps within 2 days makes it a promising strategy to try. Although 3 swaps could be deemed manageable, more research is needed to determine a point of saturation. That is, is there an optimal number of substitutions that would produce an increase in MSDP scores? If there is an optimal number of substitutions, the concept of a specific number of food substitutions could be backed by the behavior change notion of implementation intentions. Implementation intentions specify the where, when, and how of goal setting, thereby increasing the likelihood that the goal will be accomplished compared to intentions that merely specify a desired end state (52). Our study's identification of the food groups and amounts needed each simple swap outlines the specifics that may better equip a person to meet their goal of following an MSDP.

The positive effects of the honey-food pairing substitutions on MSDP scores support increased consideration of palatability in the strategy of simple swaps. The taste of some foods (e.g., vegetables and whole grains) sometimes conflicts with an innate preference for sweets (53, 54). The honey-food pairing recipes were directly informed by the isocaloric substitution modeling results. For example, olive oil was used as a base for the honey-food pairing recipes, given the magnitude of impact of the simple olive oil substitution. The ubiquity of olive oil in culinary practice and utilizing recipes that contain olive oil as a base has the potential to impact a wide audience. A potential concern was that the increase to the added sugar component would negate a positive effect on MSDP scores. The results show only a marginal difference in MSDP scores compared to the substitutions without honey. Interventions testing differences in acceptance between the simple substitutions with and without honey may be worthy of further exploration. Because our study used a nationally representative sample, the results suggest a range from 25% to 99% of if Americans in most need of improvement, could see a meaningful increase could see a meaningful increase in MSDP adherence by incorporating just 3 honey-food substitutions in their diet.

This present study has multiple strengths that support its contribution to a better understanding of dietary substitutions in large populations. To the best of our knowledge, this study is the first to use NHANES data to show the majority of Americans diets do not align with an MSDP. NHANES provides a large and reliable sample and uses a multiple-pass system for dietary recalls, creating high statistical power. The MSDP scoring system developed by Rumawas and colleagues (13) offered a number of advantages over other MSDP scoring indices that made it more appropriate for non-Mediterranean cultures throughout the world. Other studies constructed scores based on the Mediterranean diet pyramid, but did not consider the recommended intakes assigned to each food group and, thus, reflected dietary patterns of the study population rather than adherence to the MSDP (55, 56). Moreover, previous scoring indices did not consider the negative implications of overconsumption. As a result, adherence to the MSDP may have been achieved by simply consuming greater amounts of food (57). This aspect may be particularly relevant to American cohorts, where overconsumption of foods may be more common and failure to account for it may result in confounding by energy intake.

The current study's results should be considered against its potential limitations. Because there is no universally accepted scoring index to measure Mediterranean diet adherence, the limitations of the chosen system would apply to the results of current study as well. For example, in calculating the MSDP score, in order to attain the maximum of 10 for each food component, the recommended amounts of each food component were absolute values, which may not fully align with language often used regarding intake amounts set forth by dietary guidelines (e.g., “at least” or “no more than”). Olive oil as a categorical variable rather than a continuous variable would not account for over- or underconsumption, as with the other food components. The authors of the MSDP score justified this scoring pattern because the Mediterranean diet pyramid does not include a recommended amount of olive oil. As such, interpretation of the magnitude of impact of the substitution of partial olive oil for no olive oil should be considered carefully. In comparison, the simplicity of this substitution makes it favorable in terms of practicing it from a behavioral standpoint. Additional limitations of the current study include the potential recall or self-reporting biases that may arise when conducting a 24-hour recall. Additionally, the use of a 2-day diet recall may not accurately reflect dietary intakes over time. This presents a unique challenge when assessing individual dietary components that may not be consumed or used on a daily basis, such as olive oil. However, NHANES is the most representative sample of the US population; therefore, it provides a unique opportunity to assess MSDPs in Americans. Subsequently, the substitutions suggested in this study do not take into account an economic perspective, as the swaps may be more expensive than the original item or the foods may not be available in food deserts.

In conclusion, the findings of this study offer empirical evidence that most Americans are not meeting the recommendations for an MSDP. However, a few simple, isocaloric food substitutions with or without honey can help most all American adults increase alignment with to an MSDP, without sacrificing satiety. Clinicians and nutrition practitioners may find the simplicity of the message useful in promoting adherence to an MSDP.

## Data Availability

The study used all publicly available data sets: NHANES (CDC), What We Eat in America–USDA, and the USDA's Food Patterns Equivalents Database and Food and Nutrient Database for Dietary Studies.

## Notes

The authors reported no funding received for this study.

Author disclosures: FO and ACC were compensated by the National Honey Board for their time in their roles of Research Contractors, which included time conducting the study and writing the manuscript.

The National Honey Board, which operates under USDA oversight, is not a regulatory agency and does not have powers of enforcement.

Abbreviations used: DGA, Dietary Guidelines for Americans; eq, equivalents; FPED, Food Patterns Equivalents Database; HA, high-adherence group; MSDP, Mediterranean-style diet pattern; nHA, non-high-adherence group.
